# Cardiovascular magnetic resonance in wet beriberi

**DOI:** 10.1186/1532-429X-13-41

**Published:** 2011-08-12

**Authors:** Essa Essa, Michael R Velez, Sakima Smith, Shivraman Giri, Subha V Raman, Richard J Gumina

**Affiliations:** 1 Division of Cardiovascular Medicine, The Ohio State University, Davis Heart & Lung Research Institute, Suite 200, 473 W. 12th Avenue, Columbus, Ohio USA, 43210-1252; 2 Department of Internal Medicine, The Ohio State University, Davis Heart & Lung Research Institute, Suite 200, 473 W. 12th Avenue, Columbus, Ohio USA, 43210-1252

## Abstract

The clinical presentation of beriberi can be quite varied. In the extreme form, profound cardiovascular involvement leads to circulatory collapse and death. This case report is of a 72 year-old male who was admitted to the Neurology inpatient ward with progressive bilateral lower extremity weakness and parasthesia. He subsequently developed pulmonary edema and high output cardiac failure requiring intubation and blood pressure support. With the constellation of peripheral neuropathy, encephalopathy, ophthalmoplegia, unexplained heart failure, and lactic acidosis, thiamine deficiency was suspected. He was empirically initiated on thiamine replacement therapy and his thiamine level pre-therapy was found to be 23 nmol/L (Normal: 80-150 nmol/L), consistent with the diagnosis of beriberi. Cardiovascular magnetic resonance (CMR) showed severe left ventricular systolic dysfunction, markedly increased myocardial T2, and minimal late gadolinium enhancement (LGE). After 5 days of daily 100 mg IV thiamine and supportive care, the hypotension resolved and the patient was extubated and was released from the hospital 3 weeks later. Our case shows via CMR profound myocardial edema associated with wet beriberi.

## Background

Wet Beriberi is one of four clinical syndromes associated with Thiamine (Vitamin B1) Deficiency. Other clinical syndromes include Dry Beriberi, Wernicke-Korsakoff Encephalopathy, and Leigh's syndrome (Subacute Necrotizing Encephalomyopathy). Wet beriberi has varying degrees of cardiovascular involvement. In its most fulminant form, it is characterized by hypotension, tachycardia and lactic acidosis. If untreated, patients die within hours from circulatory collapse and pulmonary edema. This condition often goes unrecognized since it is easily confused with other illnesses.

## Case Presentation

A 72 year-old male was admitted to the Neurology inpatient ward with progressive bilateral lower extremity weakness and parasthesia. He carried a history of type 2 diabetes and a remote history of pancreatic cancer for which he underwent Whipple procedure. Electromyography and nerve conduction studies were consistent with symmetrical distal sensorimotor polyneuropathy. As part of his evaluation, lumbar spine magnetic resonance imaging (MRI) demonstrated enhancement of the L4-L5 and L5-S1 disks and adjacent endplates worrisome for diskitis and/or osteomyelitis. Broad spectrum antimicrobial coverage with vancomycin and ertapenem was initiated. However, CT-guided biopsy of the lumbar spine failed to show evidence of infection or inflammation.

Subsequently, the patient developed atrial fibrillation with rapid ventricular response. EKG showed ST-segment depression and T wave inversion in the precordial leads. He became hypotensive and laboratory evaluation revealed an elevated Troponin-I level of 20.95 ng/mL (Reference value < 0.11 ng/mL). He was transferred to the Cardiology Service and converted to sinus rhythm after copious IV fluid hydration and IV diltiazem infusion. Initial transthoracic echocardiography showed moderate left ventricular systolic dysfunction, normal right ventricular size and function and moderate tricuspid regurgitation.

**Figure 1:**
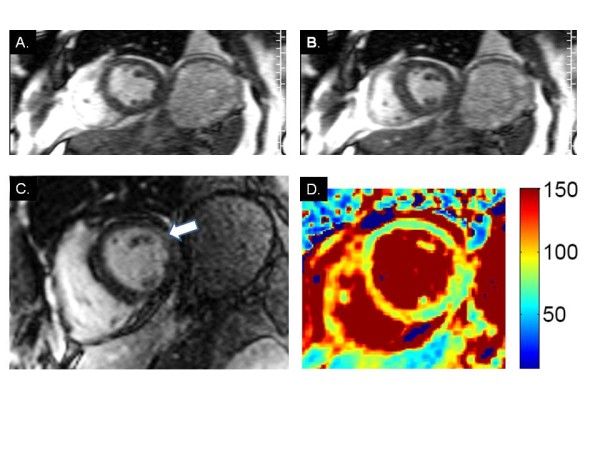
**CMR findings in wet beriberi**. End-diastolic (A) and end-systolic (B) frames from short axis cine imaging demonstrate global hypokinesis of the left ventricle. (C.) LGE image shows borderline hyperenhancement of the basal lateral wall (arrow). (D) T2 mapping demonstrates significantly elevated myocardial T2 values suggestive of diffuse edema.

The patient developed progressive ascending weakness, limited extraocular movement, confusion, edema, tachycardia and hypotension. He was intubated and mechanically ventilated. Inotropic and vasopressor support with dopamine and norepinephrine was initiated with continued IV fluid administration. Laboratory workup revealed lactic acidosis with a lactate of 7.3 mmol/L (Normal: 0.7-2.5 mmol/L) and acute renal failure. Emergent coronary angiography showed no flow-limiting coronary artery disease. Blood, urine, sputum and cerebrospinal fluid cultures were all negative. A history of chronic diarrhea and malnutrition for >3 months was obtained from family members. With the constellation of peripheral neuropathy, encephalopathy, ophthalmoplegia, unexplained heart failure, and lactic acidosis, thiamine deficiency was suspected; a thiamine level was obtained and he was empirically initiated on intravenous thiamine replacement therapy. Given the troponin elevation in the absence of acute coronary insufficiency, Cardiovascular MR (CMR) was done to assess for myocarditis. It showed severe left ventricular systolic dysfunction, estimated LV ejection fraction 25% from visual assessment of real-time cines (Figure 1, Panels A-B) (Additional File 1), and borderline basal lateral wall enhancement by LGE (Figure 1, Panel C). T2-mapping was performed using a T2-prepared steady-state free precession sequence used to generate three T2-weighted images, one each with different T2 preparation times (TE_T2P _= 0 ms, 24 ms, 55 ms) as previously described [1,2] (Figure 1, Panel D). T2-mapping suggested significant edema with T2 = 88 ms in a region of interest encompassing the entire mid-short axis myocardium.

The suspicion for thiamine deficiency was supported by an MRI of the brain showing T2-bright signal surrounding the aqueduct of Sylvius, a typical feature of thiamine deficiency. Following initiation of thiamine replacement therapy the lactic acidosis resolved twelve hours after the first dose. The pre-therapy thiamine level was found to be 23 nmol/L (Normal: 80-150 nmol/L), consistent with the diagnosis of beriberi. After 5 days of daily 100 mg IV thiamine and supportive care, the hypotension resolved and the patient was extubated and was released from the hospital 3 weeks later.

Wet Beriberi, one of four clinical syndromes associated with thiamine deficiency, is characterized by varying degrees of cardiovascular involvement. In its most fulminant form, this condition is characterized by hypotension, tachycardia and lactic acidosis. If untreated, patients die within hours from circulatory collapse and pulmonary edema [3]. This condition often goes unrecognized since it is easily confused with other illnesses.

Our patient was in shock that resolved within a few days of thiamine replacement. The diagnosis was confirmed several days later when the thiamine level value returned low. He had multiple risk factors for thiamine deficiency including a history of a Whipple procedure, malnutrition and advanced age. He developed the most severe form of beriberi "Shoshin" beriberi (from Japanese *Sho *meaning acute damage and *Shin *meaning heart), characterized by hypotension, tachycardia and lactic acidosis [4]. Thiamine is a water-soluble vitamin that serves as a cofactor for two key enzymes in the Tricarboxylic acid cycle, pyruvate dehydrogenase and α-ketoglutarate dehydrogenase. Therefore, thiamine deficiency leads to anaerobic metabolism and lactic acidosis that reverses with thiamine replacement. The mechanism of heart failure has been hypothesized to stem from reduced cellular ATP and vasodilatation [5], which may help explain the markedly elevated myocardial T2 values in this case. The marked increase in T2 may be due to dissociation of myocardial water from its protein-bound state resulting from the acidosis associated with this disorder. In our case, the clinical manifestations resolved within several days of initiation of thiamine replacement as did the metabolic acidosis.

## Conclusions

This case highlights the value of timely recognition and treatment of a potentially fatal but reversible cause for acute cardiovascular compromise. Furthermore, to the best of our knowledge, this is the first report demonstrating the CMR finding of myocardial edema associated with wet beriberi.

## Consent

Written informed consent was obtained from the patient and placed in the medical record for publication of this case report and any accompanying images. A copy of the written consent is available for review by the Editor-in-Chief of this journal.

## Competing interests

Relationships with Industry: Siemens (SVR)

## Authors' contributions

EE, MRV, SS, SVR and RJG all conceived the study, participated in the direct care and diagnosis of this patient and in the drafting of the manuscript. SVR conceived and conducted the T2 imaging study analysis to assess this patient's cardiomyopathy. SG assisted with implementation of the T2 map sequence. All authors read and approved the final manuscript.

## Supplementary Material

Additional file 1 **Cine-loop image demonstrating reduced LV systolic function**. Click here for file
